# *Mycobacterium tuberculosisβ*-Carbonic Anhydrases: Novel Targets for Developing Antituberculosis Drugs

**DOI:** 10.3390/ijms20205153

**Published:** 2019-10-17

**Authors:** Ashok Aspatwar, Visvaldas Kairys, Sangeetha Rala, Mataleena Parikka, Murat Bozdag, Fabrizio Carta, Claudiu T. Supuran, Seppo Parkkila

**Affiliations:** 1 Faculty of Medicine and Health Technology, Tampere University, FI-33014 Tampere, Finland; mataleena.parikka@tuni.fi (M.P.); seppo.parkkila@tuni.fi (S.P.); 2 Department of Bioinformatics, Institute of Biotechnology, Life Sciences Centre, Vilnius University, Saulėtekio al. 7, LT-10257 Vilnius, Lithuania; visvaldas.kairys@bti.vu.lt; 3 Tampere University of Applied Sciences, Kuntokatu 3, FI-33520 Tampere, Finland; sangeetha.rala@tuni.fi; 4 Neurofarba Department, Sezione di Chimica Farmaceutica e Nutraceutica, Università degli Studi di Firenze, Via U. Schiff 6, I-50019 Sesto Fiorentino (Firenze), Italy; bozdag.murat@unifi.it (M.B.); fabrizio.carta@unifi.it (F.C.); claudiu.supuran@unifi.it (C.T.S.); 5 Fimlab Ltd. and Tampere University Hospital, FI-33520 Tampere, Finland

**Keywords:** *Mycobacterium tuberculosis*, *β*-carbonic anhydrases, drug targets, tuberculosis, carbonic anhydrase inhibitors, in vitro inhibition, antituberculosis drugs

## Abstract

The genome of *Mycobacterium tuberculosis* (*Mtb*) encodes three *β*-carbonic anhydrases (CAs, EC 4.2.1.1) that are crucial for the life cycle of the bacterium. The *Mtb* *β*-CAs have been cloned and characterized, and the catalytic activities of the enzymes have been studied. The crystal structures of two of the enzymes have been resolved. In vitro inhibition studies have been conducted using different classes of carbonic anhydrase inhibitors (CAIs). In vivo inhibition studies of pathogenic bacteria containing *β*-CAs showed that *β*-CA inhibitors effectively inhibited the growth of pathogenic bacteria. The in vitro and in vivo studies clearly demonstrated that *β*-CAs of not only mycobacterial species, but also other pathogenic bacteria, can be targeted for developing novel antimycobacterial agents for treating tuberculosis and other microbial infections that are resistant to existing drugs. In this review, we present the molecular and structural data on three *β*-CAs of *Mtb* that will give us better insights into the roles of these enzymes in pathogenic bacterial species. We also present data from both in vitro inhibition studies using different classes of chemical compounds and in vivo inhibition studies focusing on *M. marinum,* a model organism and close relative of *Mtb*.

## 1. Introduction

Diseases caused by *Mycobacterium tuberculosis* (*Mtb*) and other mycobacterial species affect a large number of people in the world [1]. According to recent World Health Organization (WHO) estimates approximately 10 million people developed TB disease in 2017 [2]. Worldwide, tuberculosis (TB) is one of the top 10 causes of death and the leading cause of a single infectious agent. In 2017, TB disease caused approximately 1.6 million deaths [2]. In addition, drug-resistant TB continues to be a public health problem. The best estimate is that in 2017 558,000 people (range, 483,000–639,000) developed TB that was resistant to rifampicin (RR-TB), the most effective first-line drug, and 82% of these cases had multidrug-resistant TB (MDR-TB) [2].

The genome-sequencing project of *Mtb* [3] and other pathogenic bacteria including *M. marinum* (*Mmar*), a model bacterium, have greatly increased our knowledge about the evolution of mycobacteria [4,5]. Sequencing of the genomes of pathogenic mycobacteria also helped us to identify new drug targets, which might lead to the development of anti-TB drugs possessing a novel mechanism of action and thus resolve the multidrug resistance (MDR) challenge of mycobacteria that has emerged recently [3,4,5,6,7]. Among these targets, three novel proteins have been identified as carbonic anhydrases (CAs, EC 4.2.1.1).

The CA enzymes are ubiquitous in nature and belong to eight different unrelated gene families (α-, *β*-, γ-, δ-, ζ-, η-, θ-, and ι-CAs) [8,9] and are known to perform important physiologic roles in living organisms [8,9,10,11]. The CAs of vertebrates belong to the α-CA gene family, which contains multiple isoforms of the enzyme. CAs are involved in a fundamental reaction in which carbon dioxide is reversibly hydrated to generate both bicarbonate and hydrogen ions for the regulation of pH homeostasis. In addition, CAs participate in biosynthetic reactions, such as gluconeogenesis and ureagenesis in mammals. Due to their association with various diseases, α-CA enzymes are considered important targets for the design of new drugs, such as antidiuretics, antiglaucoma, antiepileptic, and anticancer agents [11]. In the recent past, CAs belonging to the *β*-CA family have emerged as potential targets for developing drugs against diseases caused by bacterial and parasitic pathogens. Indeed, several in vitro inhibition studies have shown that the *β*-CAs from pathogenic bacteria and parasitic pathogens can be inhibited by certain compounds [12,13,14,15,16,17,18,19,20,21,22,23,24]. Among these inhibitors are sulfonamides and their bioisosteres, which are the main classes of CA inhibitors that have been in clinical use for more than 50 years [25,26,27,28]. In addition, many studies have shown that pathogenic organisms are susceptible to CA inhibitors in vivo as well [9,25,29,30,31].

Analysis of *Mtb* CA sequences has revealed that *Mtb* CAs belong to the *β*-CA gene family, are encoded by genes Rv1284, Rv3588c, and Rv3273, and are denoted as *β*-CA1, *β*-CA2, and *β*-CA3, respectively [3,9,32]. The *Mtb β*-CAs are zinc-containing metalloenzymes with characteristics similar to those of many other bacterial *β*-CAs, including all the conserved amino acid residues involved in the catalytic cycle, i.e., the four zinc-binding residues Cys42, Asp44, His97, and Cys101 [9]. The three *Mtb β*-CAs have been cloned and purified, and kinetic studies of these CAs have been carried out [33,34,35,36,37]. The X-ray crystal structures of two of the *Mtb β*-CAs (Rv1284 and Rv3588c) have been resolved [36,37]. Studies conducted on mycobacterial *β*-CAs have shown that they are involved in the invasion and survival of pathogens in the host environment and are thus considered potential drug targets for developing novel anti-TB agents [9]. Several in vitro inhibition studies have shown that *Mtb β*-CAs can be efficiently inhibited by different classes of inhibitors [9]. In this review, we present the molecular data on the gene and protein sequences as well as the information on 3D structures of *Mtb β*-CAs that is available in the literature. We also review the available data on the roles of *β*-CAs in *Mtb* and nontuberculous mycobacteria (NTM), which include information on *Mmar*, a model organism for studying the physiological roles of *β*-CAs in mycobacteria and a close relative of *Mtb*. Similarly, we also discuss the data from in vitro inhibition studies using different classes of chemical compounds.

## 2. Molecular Biology

The *β*-CA genes of *Mtb* (Rv1284, Rv3588c, and Rv3273) have been cloned, recombinant proteins of these CAs have been produced, and activity and inhibition studies have been carried out [34,35,36,37]. The X-ray crystal structures of two of the enzymes (*Mtb β*-CA1 and *Mtb β*-CA2) have been resolved [36,37]. The details of the molecular characterization of the *Mtb β-CA* genes are presented in the following sections.

### 2.1. M. tuberculosis Rv1284

**Figure 1:**
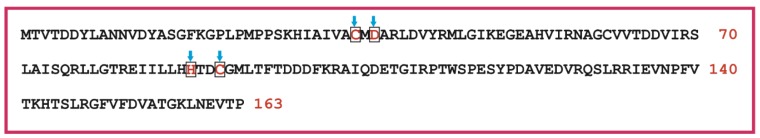
The *β*-CA sequence of *Mtb.* The protein (Rv1284) sequence shows the amino acid residues (boxed and with blue arrows) that are involved in the coordination of Zn(II) ions in the active site of the enzyme.

The gene Rv1284 encodes *Mtb β*-CA1, and the length of the corresponding peptide is 163 amino acid residues (Figure 1). The protein product of Rv1284 was identified in the membrane fraction of *Mtb* strain H37Rv by SDS-PAGE [38] and uLC-MS/MS [39]. The mRNA transcript is upregulated after 4 and 24 h of starvation, and the upregulation is highest at 96 h [40]. The gene product is required for the growth of bacteria in the C57BL/6J mouse spleen detected by transposon site hybridization (TraSH) in H37Rv [41]. The *Mtb* Rv3588c gene was cloned, and the crystal structure of the corresponding recombinant protein was studied in 2005 by Suarez Covarrubias et al. [36]. In a later report in 2009, kinetic studies of the recombinant enzyme were carried out by Minakuchi et al. (Table 1) [34]. The analysis of the cloned sequence showed no differences to the original sequence available in the UniProt database [3,34].

Interestingly, *Mtb β*-CA1 is a smaller protein compared to other *β*-CA enzymes and more specifically compared to the *β*-CA2 enzyme of mycobacteria [34]. In *β*-CA1 the Zn(II) ion is coordinated by an aspartate residue, possessing a so-called ‘closed’ active site conformation, which is also observed in some algal *β*-CA enzymes [34]. It has also been shown that many amino acids are different in *β*-CA1 compared to *β*-CA2, suggesting a structural basis for the difference in catalytic activity and affinity to CA inhibitors, as shown in Table 1 [34]. Interestingly, a study carried out in 2015 by Hofmann’s group showed that Rv1284 is susceptible to redox conditions in the host environment, linking oxidative stimuli to changes in the pH homeostasis of the pathogen [42]. The inactivation of Rv1284 due to oxidation stops the enzymatic hydration of carbon dioxide and thus the production of protons; therefore, the decrease in the pH within the vicinity of action of Rv1284 could be a strategy employed by mycobacteria for survival in the host [42].

### 2.2. M. tuberculosis Rv3588c

**Figure 2:**
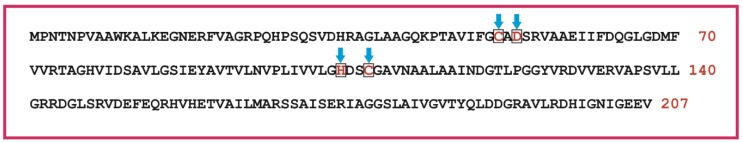
The *β*-CA2 sequence of *Mtb.* The *β*-CA (Rv3588c) protein sequence shows the amino acid residues (boxed and with blue arrows) that are involved in the coordination of the Zn(II) ion at the active site of the enzyme.

The protein encoded by gene Rv3588c, also known in the databases as CanB, is a *β*-CA and contains 207 amino acid residues (Figure 2) [36]. Biochemical experiments have shown that the protein product of Rv3588c is similar to the putative 213 aa CA Q9CBJ1 of *Mycobacterium leprae* [36]. The protein was identified by mass spectrometry in the membrane protein fraction of *Mtb* H37Rv [43] and the proteomic data supported the translation start site [44]. Transposon hybridization (TraSH) assay in H37Rv showed that the gene is required for bacterial growth in C57BL/6J mouse spleen [41].

The available data from sequence alignment and from crystallographic studies have shown that *Mtb β*-CA2 has zinc ligands that are present in other bacterial *β*-CAs [35]. Kinetic studies have shown that the product of the Rv3588c gene of *Mtb* possesses the highest catalytic activity for CO_2_ hydration among the three *Mtb β*-CAs (Table 1) [35].

### 2.3. M. tuberculosis Rv3273

**Figure 3:**
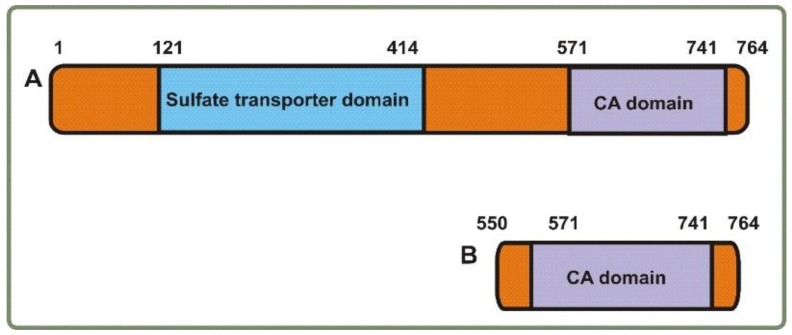
Schematic presentation of *Mtb β*-CA3 (Rv3273). (**A**) The complete *β*-CA3 protein showing both the sulfate transporter and CA domains at the N- and C-terminal ends, respectively. (**B**) The recombinant *Mtb β*-CA3 protein produced for kinetic studies [33].

The Rv3273 gene encoding *β*-CA3 was identified in 2002 from the genome of *Mtb* strain H37Rv [45]. It is believed to be a transmembrane protein based on its hydrophobic N-terminus [38,46,47]. Based on the information from UniProt and NCBI gene databases, the Rv3273 protein product is predicted to contain 764 amino acids [3,45]. It is a bifunctional protein with a sulfate transporter domain at the N-terminal end (amino acids 121–414) and a *β*-CA domain (amino acids 571–741) [33]. The schematic structure of the protein product is shown in Figure 3. The domain organization of the protein is shown to be similar to that of the other bacterial proteins found in *Arthrobacter aurescens* (YP_949116), *Leptospira borgpetersenii* (YP_798800), *Legionella pneumophila* (YP_126096), and *Leptospira interrogans* (NP_710760), which are putative bifunctional proteins and contain both sulfate transporter and *β*-CA domains [45].

The *β*-CA domain (amino acids 550–764) of the Rv3273 gene was cloned, characterized, and denominated as *β*-CA3 [33]. The amino acid sequence of the cloned DNA was shown to be similar to the amino acid sequence of Rv3273 that is available in databases [3,45] with the exception of a substitution of amino acid Arg606 to Cys. *Mtb β*-CA3 has three conserved zinc binding residues present in the *β*-CA1 and *β*-CA2 of *Mtb* [34,36,37], which are Cys584, His642, and probably Asp586 [33]. Activity and inhibition studies of *β*-CA3 have also been carried out, and the details of the kinetics are shown in Table 1.

## 3. *M. marinum* Is a Suitable Model for Studying the Roles of *β*-CAs

*M. marinum*, an NTM, is found in water and is a natural TB pathogen of zebrafish [4]. The *Mmar* bacillus has several characteristics, such as slow growth, immotility, no spores, and Gram-positive staining. *Mmar* is of great scientific interest because it is genetically related to *Mtb*, a pathogen that causes TB in humans, and interestingly, the experimental infection of *Mmar* in fish mimics the pathogenesis of TB [48]. The genome of the M strain of *Mmar* contains a 6,636,827-bp circular chromosome. *Mmar* is widely used as a model organism to study TB, and genome comparisons have confirmed the close genetic relationship between these two species showing that they share 3000 orthologs with an average amino acid identity of 85% [4].

### Sequence and Phylogenetic Analysis of β-CAs

**Figure 4:**
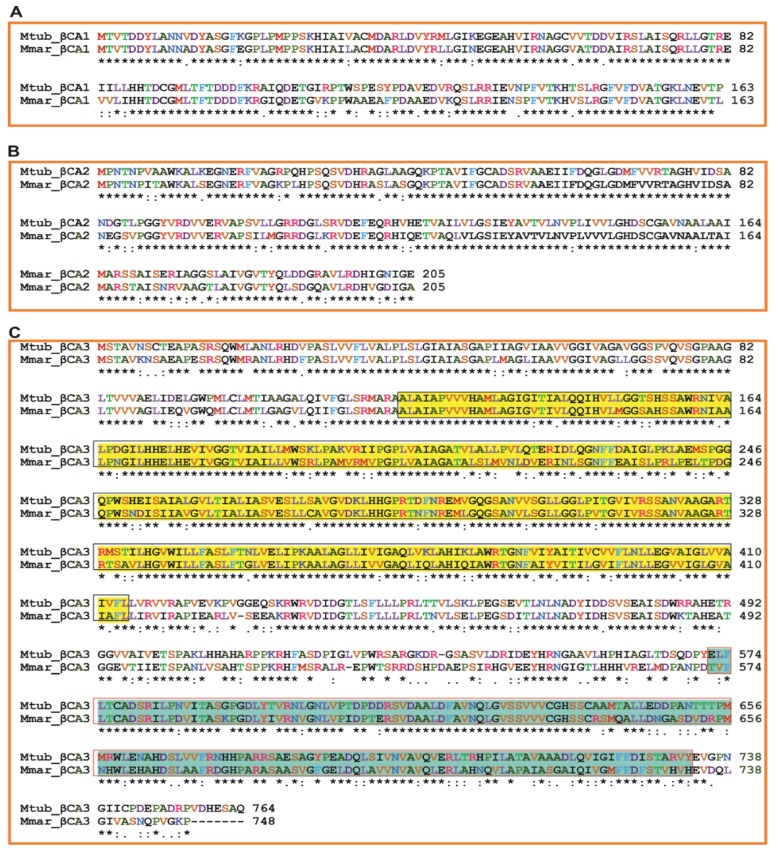
Alignment of the *Mtb* and *Mmar β*-CA protein sequences. Conserved amino acid residues between the three *Mtb* CAs (UniProt IDs: Rv1284/CA1, Rv3588c/CA2, and Rv3273/CA3) and the corresponding *Mmar β*-CA sequences are indicated by asterisks. (**A**) Shows the alignment of *β*-CA1 sequences of *Mtb* and *Mmar*; (**B**) Shows the alignment of *β*-CA2 sequences of *Mtb* and *Mmar*; and (**C**) shows the alignment of *β*-CA3 sequences of *Mtb* and *Mmar.* The boxed yellow and blue highlighted parts show the N-terminal sulfate transporter and C-terminal CA domains, respectively.

Bioinformatic studies of different *Mmar* strains showed the presence of three *β*-CAs in the bacterium [29]. Analysis of *Mmar β*-CA sequences showed that they are closely related to the *Mtb β*-CA sequences [29]. Sequence alignment of *β*-CA protein sequences from both organisms showed very high conservation of amino acids between the sequences (identities for *β*-CA1: 85.2%, *β*-CA2: 86.8%, and *β*-CA3: 72.8%). The names, numbering and sequences of the *β*-CAs were based on the UniProt entries (Figure 4).

**Figure 5:**
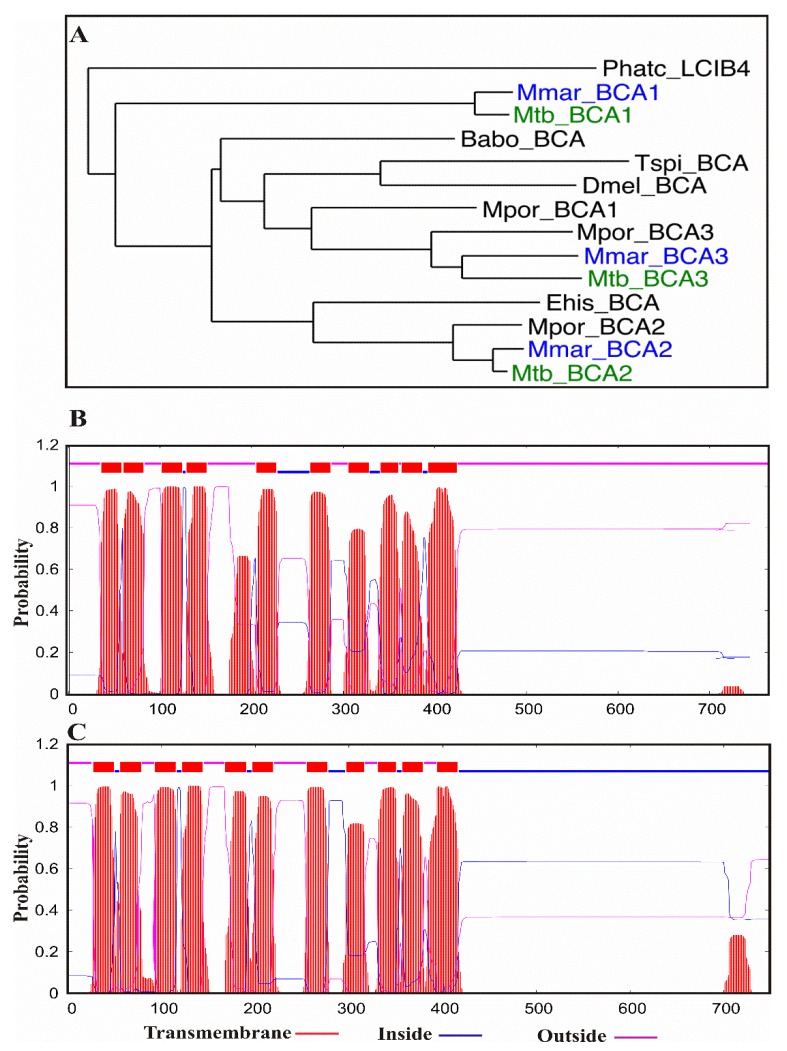
Phylogeny of mycobacterial *β*-CA sequences and transmembrane domain prediction of *Mtb* and *Mmar β*-CA3 protein sequences. The phylogenetic tree in panel (**A**) shows the evolutionary relationship of the *Mtb* and *Mmar β*-CA protein sequences in comparison with *β*-CAs from other organisms (Phatc = *Phaeodactylum tricornutum*; Babo = *Brucella abortus*; Tspi = *Trichinella spiralis*; Dmel = *Drosophila melanogaster*; Mpor = *Mycolicibacterium porcinum*; and Ehis = *Entamoeba histolytica*). Panel (**B**) shows the predicted transmembrane plot of *Mtb β*-CA3, and panel (**C**) shows the predicted transmembrane plot of *Mmar β*-CA3 [49].

Phylogenetic analysis showed that each of the three *Mmar β*-CAs are more closely related to the corresponding *Mtb β*-CA compared to the *β*-CA of other mycobacterial species (Figure 5A). A subcellular localization prediction using a web-based tool [49] (and the information accessed from the TubercuList (http://tuberculist.epfl.ch) database suggested that *Mtb β*-CA1 and *β*-CA2 are cytoplasmic (data not shown), *Mtb β*-CA3 is a membrane-associated protein (Figure 5B), and these predicted localizations match those of the *Mmar β*-CAs (Figure 5C) [49].

## 4. Structures of *Mtb β*-CAs

**Figure 6:**
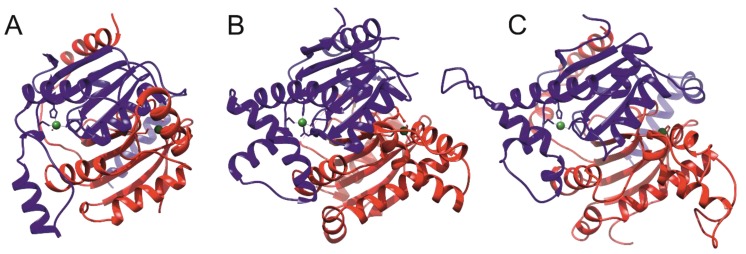
Structures of *Mtb β*-CA catalytic domains. (**A**) X-ray structure of Rv1284 (1YLK); (**B**) X-ray structure of Rv3588c (1YM3); (**C**) homology model of Rv3273 from SWISS-MODEL Repository. The two chains of the homodimers are colored blue and red. Zinc ions are colored green [36,37].

**Figure 7:**
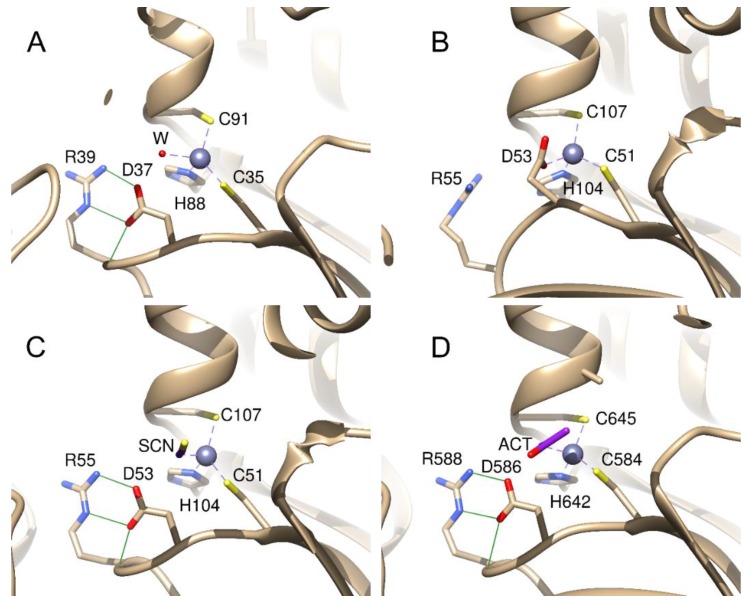
The active site of *Mtb β*-CAs. (**A**) Rv1284, PDB ID: 1YLK, (**B**) open conformation of Rv3588c, PDB ID: 1YM3, (**C**) closed conformation of Rv3588c, PDB ID: 2A5V, (**D**) homology model of Rv3273 [36,37]. Zinc is shown as a gray sphere. In the inactive conformation, Asp in the binding site ligates to Zn, and in the active conformation, Asp forms a salt bridge with Arg and a hydrogen bond with the Arg backbone N. The zinc coordination bonds are shown as dashed lines, and the hydrogen bonds between Asp and Arg are shown as green lines. In the active form, Zn is ligated to either water (**A**) or other ligands (**B**–**D**).

The X-ray crystal structures of the two *Mtb β*-CAs have been resolved [36,37]. The Protein Data Bank (PDB) contains the X-ray structures of two out of the three *Mtb β*-CAs: Rv1284 (PDB IDs 1YLK, 4YF4, 4YF5, and 4YF6) and Rv3588c (PDB IDs 1YM3 and 2A5V). The structure of the catalytic domain of Rv3273 is available as a homology model in SWISS-MODEL Repository [50]. The latter structure was generated by the automated SWISS-MODEL homology modeling pipeline based on *β*-CA Can2 from a *Cryptococcus neoformans* template (2W3N, 26.3% identity). The structures of *Mtb β*-CAs are shown in Figure 6. The catalytic domains of the three *Mtb β*-CAs possess the same *β*/α fold, which is unique to the *β*-CAs [51]; therefore, their secondary structures share many similarities, as shown in Figure 6. The most important catalytic property of the protein is the difference in the environment around the zinc in the active site. The active sites of *β*-CAs are classified into ‘active’ or ‘open’ and ‘inactive’ or ‘closed’ conformations depending on the coordination of the zinc ion [51]. *Mtb* Rv3588c was shown to switch between active and inactive conformations depending on the pH [37]; presumably the same could also hold true for the other two *Mtb β*-CAs. The environment of the zinc center of each of the *Mtb β*-CAs is shown in Figure 7. The active and inactive forms of *β*-CAs are characterized by different conformations of the Asp side chain in the active site. In the inactive form, Asp is directly coordinated to the Zn ion (Figure 7B). In the active form, Asp forms a salt bridge with a nearby Arg side chain (Figure 7A,C,D). In addition, this conformation is further stabilized by a hydrogen bond from the Asp carboxy group to the Arg backbone amide (also shown in Figure 7A, C, D). As shown in Figure 7, the switch between the two *β*-CA forms is accompanied by both an Asp side chain rotation and by a movement of the backbone on which the Asp is located. By coordinating to Zn, this residue acts as an autoinhibiting agent by not allowing water molecules to bind to zinc (cf. Figure 7B,C) thus preventing the catalytic activity. It should also be noted that the enzyme activity can be affected by environmental redox conditions leading to a chemical modification of the active site, as shown by applying redox agents to Rv1284 [42]. Oxidative conditions result in the removal of cysteine Cys35 (Figure 7A) from the Zn coordination sphere by forming a disulfide bond between Cys35 and a more remotely positioned Cys61 (not shown), which could even lead to the loss of zinc ions. It was also shown that the Tyr120 residue may be critical in the oxidative inactivation of Rv1284 [42]. Notably, in Rv3588c and Rv3273, there is no Cys in the homologous position of Cys61, implying a higher resistance to oxidative stress in these two *Mtb β*-CAs [42].

## 5. Expression Analysis of *β*-CAs from *Mmar*

**Figure 8:**
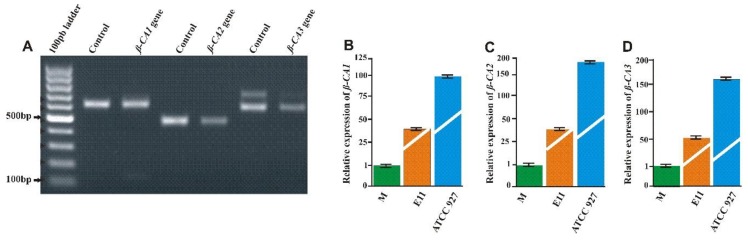
Expression analysis of *β*-CAs from *Mmar*: (**A**) The qualitative expression analysis of *Mmar β*-CA genes showed the presence of all three *β*-CAs in the ATCC 927 strain. Genomic DNA was used as a positive control. (**B**–**D**) Relative expression analysis of three *β*-CA genes from the three strains of *Mmar* using RT-qPCR [29].

Expression analysis of the three *β*-CA genes was carried out in the *Mmar* ATCC 927 strain using PCR, and the presence of transcripts was seen as bands of 500 bp for *β*-CA1, 490 bp for *β*-CA2, and 600 bp for *β*-CA3 (Figure 8A) [29]. Similarly, quantitative expression analysis of the *β*-CA genes in different strains of *Mmar* (M, ATCC 927 and E11) was also performed using RT-qPCR, and the highest expression was found in the ATCC 927 strain compared to the M and E11 strains. The molecular analysis thus confirmed the presence of all three *β*-CA genes in *Mmar (*Figure 8).

## 6. In Vitro Inhibition of Mycobacterial *β*-CAs

### 6.1. Inhibition of Mtb β-CAs Using Sulfonamides and Their Derivatives

The inhibition studies on *Mtb β*-CA1 were performed using a panel of sulfonamides, sulfamates, and their derivatives, some of which are in clinical use [34]. Most of the tested compounds showed high affinity for *β*-CA1 (inhibition range of 1–10 µM). The compounds with inhibition in the submicromolar concentration range (*K_I_* values of 0.481-0.905 µM) included sulfanilyl-sulfonamides, such as acetazolamide, methazolamide, dichlorophenamide, dorzolamide, brinzolamide, benzolamide, and the sulfamate topiramate [34]. The most efficient inhibitors (*K_I_* values of 97–186 nM) of this enzyme were 3-bromosulfanilamide and indisulam. The promising inhibition profiles of sulfonamides and their derivatives used in this study suggested that the *Mtb β*-CA1 enzyme is a potential target for developing anti-TB agents with a different mechanism of action [34].

Similarly, the inhibition profiles of a series of diazenylbenzenesulfonamides derived from sulfanilamide or metanilamide were shown to inhibit *Mtb β*-CA2 in the range of 45–955 nM [52]. Subsequently, to obtain inhibitory molecules with better inhibition properties, new compounds were synthesized by diazotization of aminosulfonamide and by coupling with phenols or amines [35]. These molecules were subsequently incorporated with various R moieties in the molecule—such as hydroxy, amino, methylamino, dimethylamino, and sulfonate—which could induce water solubility of these compounds as sodium salts. Among the molecules thus prepared, the aminomethylene sodium sulfonate derivatives and their corresponding N-methylated analogues showed the most efficient inhibition properties (*K_I_s* of 45–59 nM) [35]. Later, the diazenylbenzenesulfonamides were also tested for the inhibition of *Mtb β*-CA3, and among them, prontosil was found to be the best inhibitor (*K_I_* 126–148 nM) [53]. In another study, several compounds were tested for their inhibition efficiency against *Mtb β*-CA3. Compounds that inhibited the activity of this enzyme in the nanomolar range included 2-amino-pyrimidin-4-yl-sulfanilamide (*K_I_* 90 nM) and sulfonylated sulfonamide (*K_I_* of 170 nM). These studies suggested that *Mtb β*-CA3 could be targeted using CAIs with the potential to develop agents that target *Mtb* [33].

The sulfonamides prepared by reaction of sulfanilamide with aryl/alkyl isocyanates showed inhibition of *Mtb β*-CA1 and *β*-CA3 in the range of 4.8–6500 nM and 6.4–6850 nM, respectively. The structural activity relationship related to inhibition has been shown to be associated with the nature of the moiety substituting the second ureido nitrogen, and it is the determining factor in controlling the inhibitory power. This may be due to flexibility of the ureido linker and the ability of this moiety to orientate in different subpockets of the active site cavities of these enzymes [54]. The inhibition of all three *Mtb β*-CAs using a number of halogenated sulfanilamides and halogenated benzolamide derivatives showed inhibition efficiency in the submicromolar to micromolar range. The substitution pattern at the sulfanilamide moiety/fragment of the molecule is crucial for the inhibition efficiency of the molecule. The best inhibitors were the halogenated benzolamides (*K_I_s* in the range of 0.12–0.45 μM), whereas the halogenated sulfanilamides were slightly less inhibitory (*K_I_s* in the range of 0.41–4.74 μM) [55]. Similarly, the inhibition of the three *Mtb β*-CAs was carried out using a series of fluorine-containing sulfonamides that were modified with amino, amino alcohol, and amino acid moieties [56]. Among these, some showed efficient inhibition of *β*-CA2 (*K_I_* values in the nanomolar range) and moderate inhibition efficiency against *β*-CA1 and *β*-CA3 (*K_I_* in the low micromolar range) [56]. Recently, a novel sulfonamide molecule obtained from sulfanilamide, which was N4-alkylated with ethyl bromoacetate followed by a reaction with hydrazine hydrate and further reacted with various aromatic aldehydes, showed *K_I_s* in the range of 127 nM–2.12 μM for *Mtb β*-CA3 [57].

### 6.2. Mono and Dithiocarbamates as Inhibitors of Mycobacterial β-CAs

Studies have shown that both mono-(MTCs) and dithiocarbamates (DTCs) selectively inhibit *Mtb β*-CAs. The inhibition of *Mtb β*-CA1 and *β*-CA3 using a series of *N*-MTCs and *N,N*-disubstituted DTCs showed inhibition efficiencies in the subnanomolar to the micromolar range [58]. The inhibition properties of these compounds were dependent on the substitution pattern at the nitrogen atom of the DTC zinc-binding group. Replacement with aryl, arylalkyl-, heterocyclic as well as aliphatic and aminoacyl moieties led to potent *β*-CA1 and *β*-CA2 inhibitors in both the *N*-mono-and *N,N*-di-substituted DTC series [58].

### 6.3. Phenolic Natural Products and Phenolic Acids as Mycobacterial β-CA Inhibitors

In search of novel inhibitors that could selectively inhibit *Mtb β*-CAs, a series of phenolic natural products (NPs) were screened against *Mtb β*-CA1 and *Mtb β*-CA3. It was shown that NPs were able to selectively inhibit *Mtb β*-CA1 and *β*-CA3 with *K_I_s* in the submicromolar range [59]. Similarly, screening of a series of phenolic acids and their derivatives against all three *Mtb β*-CAs showed good inhibition efficiencies (*K_I_s* 1.87 μM–6.09 μM). In addition, these compounds showed no inhibitory activity against human CA I or CA II, suggesting that the phenolic compounds could potentially be developed as anti-TB agents [60]. Computational analysis of the binding mode of the compounds suggested that the inhibitors anchored to the zinc-coordinated water molecule in the CA active site and interfered with the nucleophilic attack of the zinc hydroxide on the substrate CO_2_ [60]. Screening of a series of C-cinnamoyl glycosides containing the phenol moiety against the three *Mtb β*-CAs also showed that most of the compounds were highly efficient inhibitors of *Mtb β*-CA2 (*K_I_* 130–940 nM), showing a preference for *β*-CA2 over human CA II [61].

### 6.4. Carboxylic Acids as Inhibitors of Mycobacterial β-CA

Carboxylic acids are known to inhibit *Mtb β*-CAs, but the inhibitory mechanism of these acids is not known. Screening of scaffolds containing carboxylic acids, such as benzoic acid, nipecotic acid, ortho and para coumaric acid and ferulic acid, against all three *Mtb β*-CAs showed inhibition efficacy in the micromolar range (*K_I_* 0.11–0.97 µM). The *K_I_*s for the inhibition of *β*-CA2 were in the range of 0.59–8.10 µM, whereas against *β*-CA1, the carboxylic acids showed inhibition constants in the range of 2.25–7.13 µM [62]. This class of inhibitors has not been well explored, and further in vitro inhibition studies are needed to document the real potential of these inhibitors as anti-TB agents.

## 7. Inhibition of Mycobacterial Strains in Culture

In addition to studies on the in vitro inhibition of *Mtb β*-CAs, CA inhibitors have been used for the inhibition of *Mtb* growth in culture [9]. Studies using 6-mercaptopurine with sulfony/sulfenyl halides known as 9-sulfonylated/sulfenylated-6-mercaptopurines showed that the compounds inhibited growth of the wild-type *Mtb* bacilli H37Rv in the range of 0.39–3.39 μg/mL [63]. Among these compounds, one of the derivatives showed a minimal inhibitory concentration (MIC) of approximately 1 mg/mL against several drug-resistant *Mtb* strains [63]. The compounds with MICs below 1 μg/mL were considered excellent leads [63].

The inhibition of *Mmar*, a model mycobacterium, was recently investigated in liquid cultures using DTCs Fc14-584a and Fc14-584a. The DTCs were prepared by the reaction of a corresponding amine with carbon disulfide in the presence of a base. The obtained compounds were shown to be specific inhibitors of *Mtb β*-CA1 and *β*-CA3 [58]. This study showed that the minimum concentration (MIC) required for complete inhibition of *Mmar* was 75 μM. Further studies using the concentration below MIC (dilution 1:4) have shown no growth of *Mmar*, suggesting that the DTCs are bactericidal [29]. These studies demonstrate that CAIs, in addition to inhibiting the activity of mycobacterial *β*-CAs, impair the growth of both *Mtb* and NTM in culture and have significant potential as antimycobacterial compounds.

## 8. In Vivo Inhibition of Mycobacteria by CA Inhibitors

In vivo studies have shown that 6-ethoxy-1,3-benzathiazole-2-sulfonamide (ETZ) impairs the signaling of PhoPR in *Mtb* [64,65]. ETZ is a sulfonamide compound that is a general inhibitor of CA activity and is an FDA-approved drug used in the treatment of glaucoma, epilepsy, and duodenal ulcers and is also a diuretic [9]. ETZ treatment of *Mtb* induces phenotypes similar to the *PhoPR* mutants downregulating the PhoPR regulon, a reduction in virulence-associated lipids, and inhibition of Esx-1 protein secretion [64] (Table 2). Further studies on single-cell imaging of a PhoPR-dependent fluorescent reporter strain showed that ETZ inhibited PhoPR-regulated genes in infected macrophages and in mouse lungs [64]. A mouse model infected with bacteria containing a fluorescent reporter showed a reduction in bacteria in the lungs and significantly reduced GFP fluorescence compared to the control group [64].

The extracellular DNA (eDNA) in NTM is responsible for the phenotypic resistance of the bacteria to antibiotics and plays an important biological role in the bacteria [66]. Studies have shown that bicarbonate ion helps in the export of eDNA in NTM, and it is well established that the enzymatic activity of CAs is involved in the production of bicarbonate ions via the hydration of carbon dioxide [11,67]. Furthermore, it has been demonstrated that a mutant *M. avium* in which CA genes were inactivated was unable to export eDNA [67]. However, when the CA genes were restored, the transport of eDNA was also restored, suggesting that CAs play important roles in the transport of eDNA and the formation of biofilms by the bacteria [67] (Table 2). The surface-exposed proteome of *M. avium* in eDNA-containing biofilms showed that CAs are abundantly present, and the inhibition studies showed a significant reduction in eDNA transport in the presence of ETZ [67].

**Figure 9:**
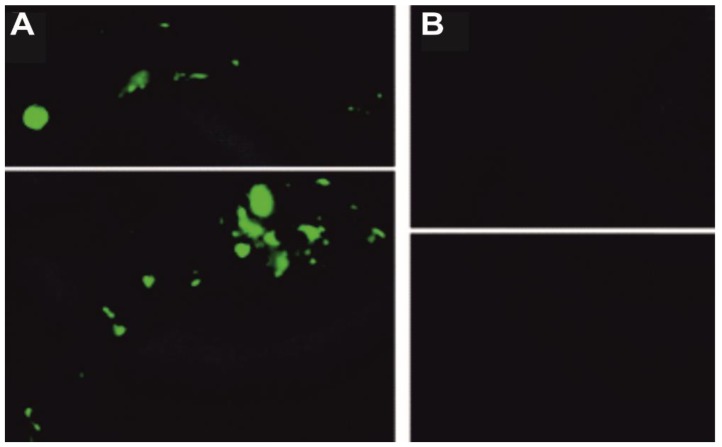
Inhibition of *Mmar* in a zebrafish model. (**A**) Green fluorescence showing the infection in zebrafish larvae at six days post infection not treated with the inhibitor; and (**B**) zebrafish larvae infected with *Mmar* and treated with 300 μM Fc14-584b showing the absence of bacteria. The figure is modified from Aspatwar et al. [29].

DTCs, another class of compounds that strongly inhibit *Mtb β*-CAs in vitro, have been used in vivo for the inhibition of *Mmar* [29,58]. The in vivo studies have shown that a DTC, FC14-384b (Figure 9B and Table 2), significantly impaired the growth of *Mmar* in zebrafish larvae [29]. The zebrafish larvae infected with the *Mmar* wasabi strain with an infection dose of 471 ± 143 (CFUs) bacteria and treated with 300 μM FC14-384b showed a significant reduction (*p* > 0.0096) in bacterial load (Figure 9B) compared to the zebrafish larvae not treated with the inhibitor (Figure 9A). These studies suggest that the compounds that selectively inhibit the activity of *β*-CAs could be useful as a new class of antimycobacterial agents that can potentially treat MDR-TB.

## 9. Conclusions

The experimental data on the biochemical and molecular characterization of *Mtb β*-CAs have provided us with better insights into the diverse roles of these enzymes in the *Mtb* pathogen. In addition to their role in the hydration of CO_2_ required for pH homeostasis in the pathogen, these enzymes have been implicated in several nonenzymatic functions that are required for the survival of *Mtb* and pathogenesis of TB disease in the host. However, precise physiological roles of the individual enzymes have yet to be discovered. In vitro inhibition studies using different classes of inhibitors have shown that *Mtb β*-CAs are druggable targets with potential as anti-TB agents with a diverse mechanism of action compared to the drugs that are in clinical use and to which the *Mtb* strains have developed various degrees of resistance.

Interestingly, recent studies have shown that CA inhibitors that selectively inhibit *Mtb β*-CAs in vitro also inhibit the growth of *Mmar* in vivo in zebrafish. Similarly, ETZ, a general CA inhibitor and a clinically used drug, inhibits the secretion of virulence factors and attenuates *Mtb*. These findings represent an in vivo proof of concept that the *Mtb β*-CA enzymes are indeed promising targets for developing antimycobacterial agents. However, we need to design novel CA inhibitors that are not only selective against the mycobacterial *β*-CAs but are also membrane permeable and safe for clinical use in humans when tested in strict preclinical and clinical settings.

## Figures and Tables

**Table 1:** Activity and inhibition kinetics of *Mtb β*-CAs compared to human CA II.

CAs	Gene ID	Protein	*K*_cat_ (S^−1^)	*K*_cat_/K_m_ (M^−1^S^−1^)	*K*_I_ (nM) ^a^	Ref.
*Mtb β*-CA1	Rv1284	163 aa	3.9 × 10^5^	3.7 × 10^7^	480	[34]
*Mtb β*-CA2	Rv3588c	207 aa	9.8 × 10^5^	9.3 × 10^7^	9.8	[35]
*Mtb β*-CA3	Rv3273	764 aa ^b^	4.3 × 10^5^	4.0 × 10^7^	104	[33]
hCA II	*CA*2	260 aa	1.4 × 10^6^	1.5 × 10^8^	12	[35]

^a^ Inhibition using acetazolamide; ^b^ Sulfate transporter domain (amino acids 121–414).

**Table 2:** In vivo inhibition studies showing the effects of CA inhibitors.

CA Inhibitor	Mycobacterium	Group	Effect on the Bacterium	References
*Ethoxzolamide*	*Mtb*	MTC	Inhibits PhoPR regulon, attenuates virulence	[64]
Ethoxzolamide	*M. avium*	NTM	Reduces the transport of eDNA and biofilm formation	[67]
Dithiocarbamate	*Mmar*	NTM	Impairs growth of *Mmar* in zebrafish larvae	[29]

## Abbreviations

The following abbreviations are used in this manuscript:

CA	Carbonic anhydrase
*Mtb*	*Mycobacterium**tuberculosis*
*Mmar*	*Mycobacterium**marinum*
CAI	Carbonic anhydrase inhibitor
MDR-TB	Multidrug-resistant tuberculosis
WHO	World Health Organization
TB	Tuberculosis
MTBC	*Mycobacterium**tuberculosis* complex
NTM	Nontuberculous mycobacteria
ETZ	Ethoxzolamide
*β*-CA1	Beta-carbonic anhydrase 1
*β*-CA2	Beta-carbonic anhydrase 2
*β*-CA3	Beta-carbonic anhydrase 3
RR-TB	Resistant to rifampicin *Mycobacterium* *tuberculosis*
eDNA	Extracellular DNA
PDB	Protein Data Bank
MTC	Monothiocarbamates
DTC	Dithiocarbamates
NP	Natural products
MIC	Minimal inhibitory concentration
